# A Retrospective Quantitative Analysis of the Shared Care Glaucoma Scheme (SCGS) in Monitoring Glaucoma Progression During COVID-19

**DOI:** 10.7759/cureus.80190

**Published:** 2025-03-07

**Authors:** Risantini Murugan, Rui Shian Lee, Hariss G Paremes Sivam, Pankaj K Agarwal

**Affiliations:** 1 Oncology, Queen Elizabeth Hospital Birmingham, Birmingham, GBR; 2 Psychiatry, Royal Infirmary of Edinburgh, Edinburgh, GBR; 3 Cardiology, Oxford University Hospitals National Health Service (NHS) Foundation Trust, Oxford, GBR; 4 Ophthalmology, The Princess Alexandra Eye Pavilion, Edinburgh, GBR

**Keywords:** community service, covid-19, glaucoma practice, glaucoma progression, ophthal, optometrist

## Abstract

Objective: This study aimed to investigate the impact of the shared care glaucoma scheme (SCGS), implemented during COVID-19 due to reduced clinic space and lockdown measures, on glaucoma disease progression in patients.

Background: The study was conducted between January 2021 and December 2023 and explored joint care provided by ophthalmologists and community optometrists in Lothian.

Methods: Data collected include patient demographics, intraocular pressure (IOP) readings, mean deviation visual field scores (MDVFs), a history of interventions during the pre-SCGS phase, glaucoma medications, and referrals to Princess Alexandra Eye Pavilion (PAEP). The IOP, MDVF, and glaucoma medications were collected at three stages: pre-SCGS, SCGS, and post-SCGS. The Glauc-Strat-Fast tool was used to assign each patient a pre-SCGS glaucoma stage.

Results: A total of 172 patients (85 males, 49.4%) participated in this study, with a mean age of 77.7 years (SD = 10.4). Sixteen patients (9.30%) were referred to PAEP during the scheme; the Red group of patients, identified using the Glauc-Strat-Fast tool, represented five of these referrals (31.3%). Four of the referred patients (25%) had an intervention at PAEP, either a change in medication or a surgical procedure. There was no significant increase in the IOP values from the pre-SCGS phase to the post-SCGS phase. The MDVF values had a mean deterioration of 0.5998 dB from the pre-SCGS phase to the post-SCGS phase.

Conclusions: Overall, disease stability was maintained in all glaucoma subgroups. The study, therefore, suggests the possibility of implementing the SCGS in the United Kingdom as part of routine glaucoma service in the post-COVID-19 era.

## Introduction

Glaucoma is a chronic eye condition where an absolute or relative increase in intraocular pressure (IOP) causes damage to the optic nerve and deterioration in vision [1-3]. The relative increase in IOP refers to levels within the standardized normal range but still considered high for some patients, causing blindness. Several studies identified IOP as the only modifiable risk factor in preventing the deterioration of vision. These studies also emphasize the significance of timely interventions to manage glaucoma progression [4,5]. It is one of the major causes of blindness worldwide and is known to be more detrimental compared to cataracts due to its irreversible nature [6]. Periodic monitoring of the IOP, optic disc, and visual field is crucial to allow timely management and intervention in this condition due to the silent progressive nature of the condition [3].

The COVID-19 pandemic has had a detrimental effect on the treatment and follow-up of glaucoma on a global scale. Due to the fear of spreading infection, multiple-phased lockdowns were imposed, thus disrupting glaucoma follow-up care models in various countries. The main challenges in periodic follow-ups during this period were transportation restrictions, socioeconomic factors, and reduced clinic space and staff at hospital eye services (HES) [7]. In response to the pandemic, alternative models were proposed to ensure the continuity of care in glaucoma patients. In Lothian, Edinburgh, the challenge was overcome by launching a shared care glaucoma scheme (SCGS), which involved appointing National Health Service (NHS) Education for Scotland Glaucoma Award Training (NESGAT)-qualified optometrists to conduct an interval follow-up at the community optometrist in between HES clinic appointments. The SCGS was launched and piloted for a year beginning in February 2020 [8]. However, there is a lack of research into the scheme's impact on glaucoma progression.

This study aims to investigate the impact of the SCGS in this group of glaucoma patients. It aims to stratify this patient group based on the Glauc-Strat-Fast tool and evaluate the progression in IOP values and visual field effects. By taking into consideration the individual patient risk factors (analysis of visual field mean deviation and rate, IOP, ophthalmic comorbidities, and systemic and social factors) and their specific risk of progression, the Glauc-Strat-Fast tool recommends to us the appropriate clinical qualification of the team member supervising the patient. An optometrist or ophthalmologist can monitor patients in the G1-G3 and A1-A3 categories, and clinical data can be captured and reviewed again later for decisions. Patients in R1-R3 categories have red flags, and a consultant-led inpatient review is required for monitoring. This helps the team appropriately direct available team members to ensure that patient caseloads are managed in a timely, safe, and effective manner. This study will guide healthcare professionals, especially those involved directly in following up with glaucoma patients, in modifying and continuing the SCGS in the future. Additionally, the possibility of implementing this scheme in other regions in the United Kingdom can be evaluated.

## Materials and methods

Study design and setting

This was a retrospective cohort study conducted at the Princess Alexandra Eye Pavilion (PAEP), Edinburgh. The study aimed to evaluate the impact of reduced frequency of glaucoma clinic attendance at a hospital eye service (HES) by chronic glaucoma patients who had participated in the Lothian SCGS in 2021. We aimed to investigate the proficiency of SCGS in flagging patients with concerning features of progression (increasing IOP and mean deviation visual field score, MDVF). Data were extracted from hospital medical records through paper notes at PAEP.

Inclusion and exclusion criteria

A total of 266 patients were screened for inclusion in the study. The inclusion criteria included patients who had participated in SCGS from January 2021 to December 2023 and were risk-stratified according to the Glauc-Strat-Fast tool. Patients were excluded due to nonattendance at clinic appointments and if the clinical records were incomplete. Based on the criteria, 94 (35.3%) patients were excluded from the study, and a final number of 172 (64.7%) patients were included.

Data collection

Patient data were obtained from hospital medical records at PAEP and organized on an Excel Sheet (Microsoft Corporation, Redmond, WA). Data collected included age, gender, initial presentation at PAEP, diagnosis, IOPs, mean deviation of visual fields, gonioscopy, lens status, presence of intervention before participation in SCGS, evidence of referral during SCGS, ophthalmology comorbidities, and management. For each patient, the IOPs, mean deviation of visual fields, and management were recorded at baseline, and follow-up data were retrieved during and after SCGS. The IOPs and MDVF were measured at PAEP using the Goldmann applanation tonometry and Humphrey visual field analyzer at baseline and post-SCGS. During SCGS, they were measured at six different community optometrists in Lothian (KD Wallace Eyecare, Specsavers, Black and LIzars, Cameron Optometry, Rifkind and Brophy, and Montgomery Optometry).

Data analysis

Statistical analysis was performed using RStudio (version 2023.12.1+402; Posit, Boston, MA), with significance defined as p < 0.05. Descriptive and summary statistics, including mean and standard deviation (SD), were used to summarize the distribution of age and gender. The glaucoma stage is based on the Glauc-Strat-Fast tool and the trends in IOPs and MDVFs. Comparisons between the IOPs and MDVF at the three stages were made using a t-test, with a significance level set at p < 0.05. A stepwise regression model was used to assess factors that are associated with the progression of glaucoma. A threshold of 1 dB over a year was used to indicate significant deterioration. Referral rates by glaucoma subgroups were obtained.

Ethical considerations

The project was registered formally with guidance from the NHS Lothian Quality website [9]. Caldicott approval was obtained, and a project charter was completed and submitted to the Quality Improvement Team Chair [10].

Ethical approval for the study was obtained through the Caldicott approval. All patient data were collected and processed in line with the Data Protection Act 2018 to ensure the secure handling of patient information. We ensured that only one patient's identifiable information was recorded to enable unique identification, and the rest of the data were completely anonymized.

## Results

Overview of the cohort

A total of 172 patients, comprising 85 men (49.4%) and 87 women (50.6%), were included in this study, with a mean age of 77.7 years (SD = 10.4). The glaucoma stages in this patient cohort have been classified with the aid of the Glauc-Strat-Fast tool [11]. This system indicates the lifetime risk of visual loss, with the lowest risk in the Green (G) group and the highest in the Red (R) group. Within each color category, a higher number denotes an increased risk. Most patients were categorized under stage R1 (26.7%), followed by the A1 and G3 stages. The demographics and distribution of the glaucoma stages are shown in Table 1.

**Table 1:** Demographic and clinical characteristics of the study population (n = 172) Data are presented as n (%). Glaucoma stages were classified using the Glauc-Strat-Fast tool: Green 1 (G1), Green 2 (G2), Green 3 (G3), Amber 1 (A1), Amber 2 (A2), Amber 3 (A3), Red 1 (R1), and Red 2 (R2). This system indicates the lifetime risk of visual loss, with the lowest risk in the Green group and the highest in the Red group. Within each color category, a higher number denotes increased risk

Characteristic	n (%)
Sex	
Male	85 (49.4)
Female	87 (50.6)
Glaucoma stage (Glauc-Strat-Fast classification)	
G1	6 (3.5)
G2	27 (15.7)
G3	33 (19.2)
A1	44 (25.6)
A2	5 (2.91)
A3	4 (2.33)
R1	46 (26.7)
R2	7 (4.1)

Primary outcomes

**Figure 1:**
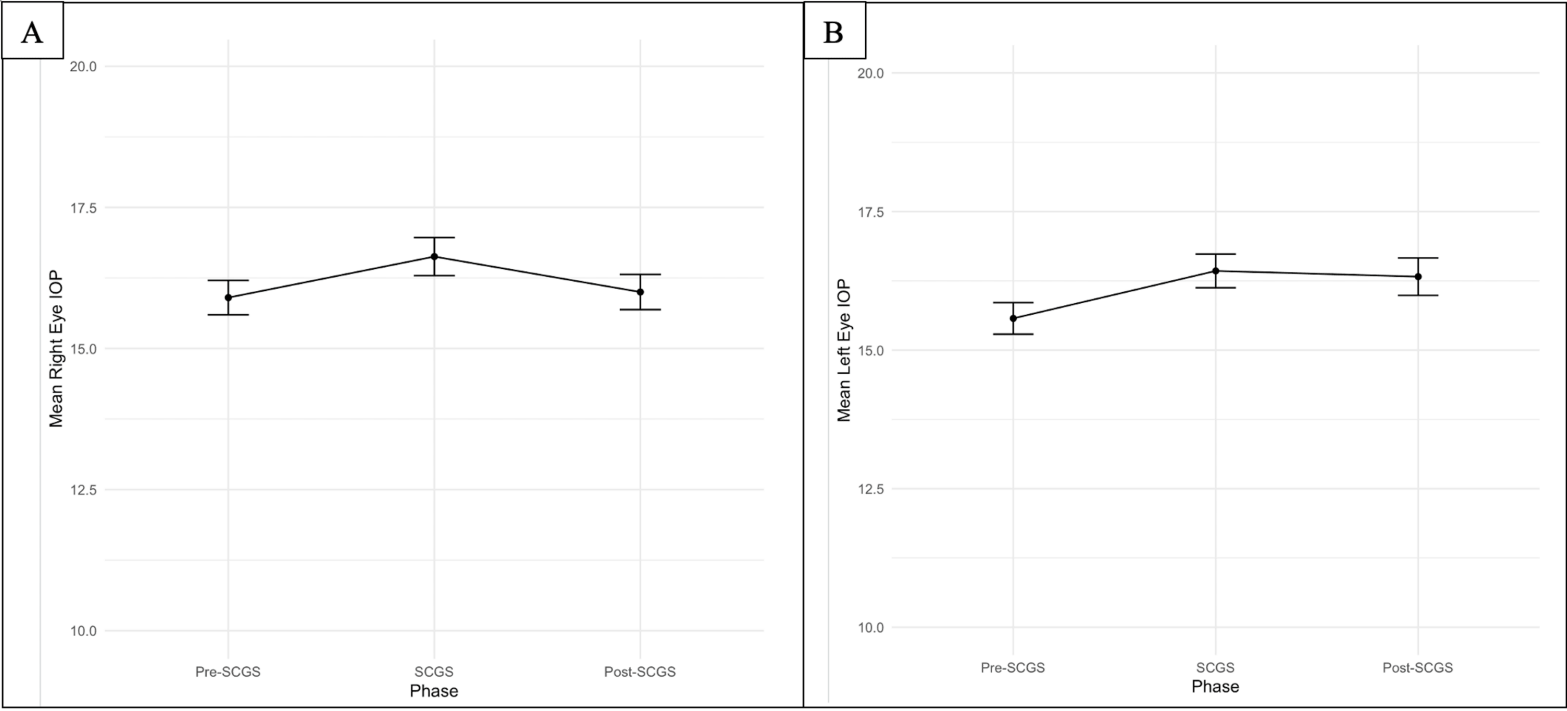
Line plots demonstrating the mean IOP in the (A) right and (B) left eyes over each phase Data are presented as mean values (black dot) ± standard error of the mean IOP: intraocular pressure; SCGS: shared care glaucoma scheme

The IOPs in both eyes showed a statistically significant increase (right eye, RE; p = 0.01167; left eye, LE; p = 0.00099) during the pre-SCGS phase when compared to the SCGS phase. The IOPs stabilized during the SCGS phase and the post-SCGS phase. RE IOP had a mean increase of 0.10 (p = 0.7644), and LE IOP had a mean increase of 0.75 (p = 0.01881) from the SCGS phase to the post-SCGS phase. Overall, there were no significant changes in the RE IOP but a statistically significant small increase in the LE IOP. Figure 1 presents a line plot demonstrating the changes in IOP in both eyes across the three phases.

Secondary outcomes

**Figure 2:**
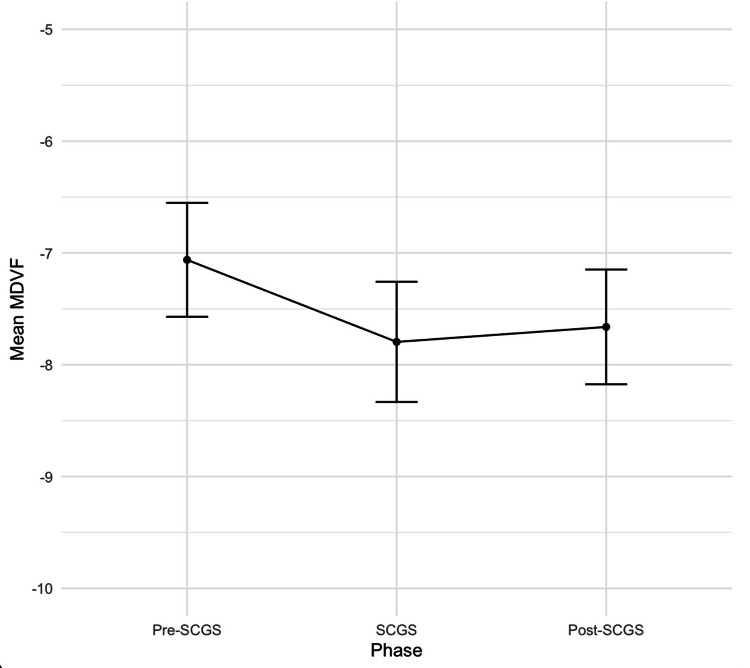
Line plot demonstrating the progression of mean of the MDVF across the pre-SCGS, SCGS, and post-SCGS phases Data are presented as mean values (black dots) ± standard error of the mean MDVF: mean deviation of the visual field; SCGS: shared care glaucoma scheme

The MDVF values experienced an overall mean deterioration of 0.7339 (p = 0.002946) from the pre-SCGS phase to the SCGS phase. From the SCGS and post-SCGS phases, the MDVF values had a mean increase of 0.1341 (p = 0.5992). Overall, from the pre-SCGS phase to the post-SCGS phase, there was a mean deterioration in MDVF values of 0.5998 (p = 0.04085). Figure 2 presents the overall trend in the mean MDVF values across the three phases for the entire cohort of 172 patients without differentiating between the patient subgroups.

Referral rates

**Figure 3:**
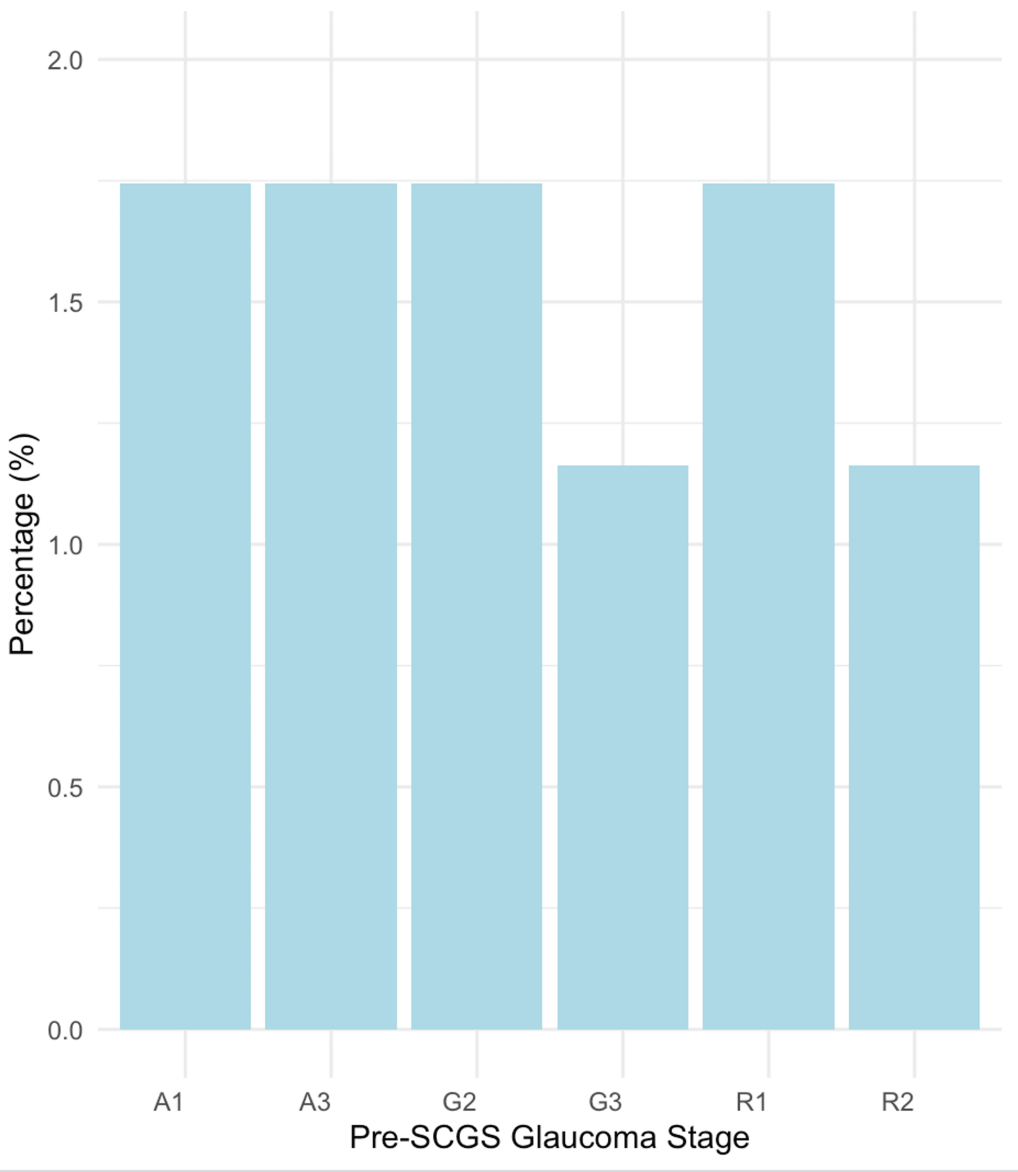
Percentage of glaucoma stages among referred cases The bar graph illustrates the proportion of glaucoma stages of the 16 patients that were referred to HES during the SCGS phase, presented in a percentage format HES: hospital eye services; SCGS: shared care glaucoma scheme; A1: Amber 1; A3: Amber 3; G2: Green 2; G3: Green 3; R1: Red 1; R2: Red 2

Sixteen patients (9.30%) were referred to HES during the SCGS phase. The proportion of glaucoma stages among the referred cases is shown in Figure 3. Among the 16 referred cases, nine were diagnosed with primary glaucoma and seven with secondary glaucoma. The primary glaucoma cases included primary open-angle glaucoma, normal pressure glaucoma, and ocular hypertension. The secondary glaucoma cases included pseudoexfoliative glaucoma, pigment dispersion glaucoma, and rubeotic glaucoma. Stages A1, A3, G2, and R1 each had three referred patients (1.74%), while stages G3 and R2 each had two referred patients (1.16%). Twelve patients (6.98%) had no interventions done at HES, and four patients (2.32%) had interventions such as bilateral selective laser trabeculoplasty, bilateral peripheral iridotomies, unilateral trabeculectomy, and the addition of an IOP-reducing agent. Table 2 shows the interventions completed at the HES for the four patients and their respective glaucoma stages.

**Table 2:** Glaucoma stages and interventions completed after referral. Glaucoma stages are Amber 3 (A3), Green 2 (G2), Red 1 (R1), and Amber 3 (A3) HES: hospital eye services; IOP: intraocular pressure

Intervention at HES	Glaucoma stage
Bilateral selective laser trabeculoplasty	A3
Bilateral peripheral iridotomies	G2
Unilateral trabeculectomy	R1
Addition of an IOP-reducing agent	A3

**Figure 4:**
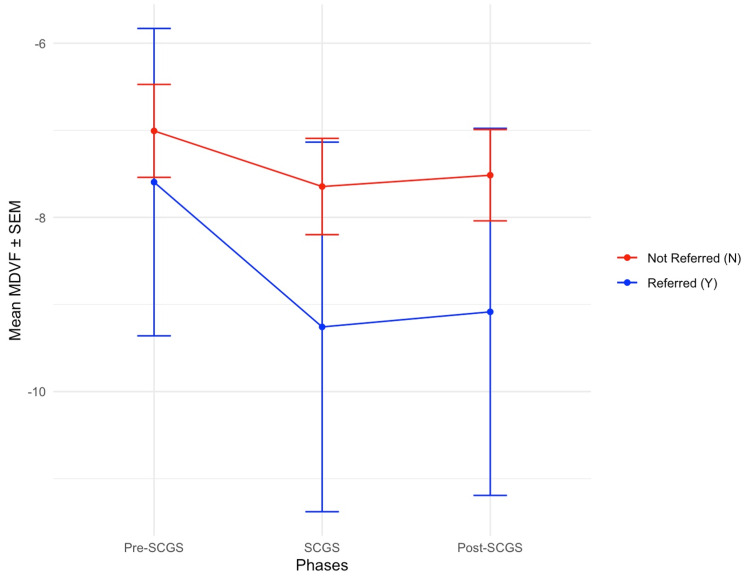
MDVF changes across phases of the referred and not referred patients Line plots demonstrate the progression of the mean of MDVF across the pre-SCGS, SCGS, and post-SCGS phases for the Referred (Y) group and the Not referred (N) group. The Referred (Y) group has been plotted in a blue line and the Not referred (N) group in a red line. Data are presented as mean values ± SEM, where blue dots represent the Referred (Y) group and red dots the Not referred (N) group MDVF: mean deviation of visual field; SCGS: shared care glaucoma scheme; SEM: standard error of the mean

Referred patients had more negative MDVF values across the three phases than those not referred. The mean MDVF values were stable (<1 dB change across the three phases) in both groups. Table 3 and Figure 4 demonstrate the comparison of mean MDVF values between patients who were referred to HES and those who were not. Table 3 presents the mean MDVF values and their associated SD values across the three phases, while Figure 4 illustrates the mean MDVF values with their respective standard errors.

**Table 3:** Mean of MDVF and SD of the MDVF values of the Referred (Y) group and the Not referred (N) group. p values of the MDVF values comparing the referred and not referred groups are displayed MDVF: mean deviation of visual field; SD: standard deviation; SCGS: shared care glaucoma scheme

Phase	Referred (Y) group		Not referred (N) group		p values
	Mean MDVF	SD MDVF	Mean MDVF	SD MDVF	
Pre-SCGS	-7.6	7.06	-7.01	6.66	0.7385
SCGS	-9.26	8.49	-7.65	6.9	0.3855
Post-SCGS	-9.08	8.43	-7.52	6.55	0.3762

Of the 156 (90.7%) patients not referred, nil significant ophthalmologic sequelae were recorded, and no statistically significant increase in IOP and MDVF was noted. This analysis implies that patients were appropriately referred to the HES during the SCGS phase based on the Scottish Intercollegiate Guidelines Network 144 guideline. This ensured that no high-risk cases were missed among patients adhering to clinic appointments.

Additional analysis

Multiple regression analysis found that male glaucoma patients (p = 0.0630) with a pre-SCGS glaucoma stage of R^2^ (p = 0.0661) had the most significant association with deterioration in MDVF values. No significant associations were found between the glaucoma stage, the presence of an intervention before SCGS, other age groups, or MDVF deterioration. The p values of the predictors analyzed are shown in Table 4.

**Table 4:** Predictors and their p values Predictors include specific age groups (40-60 and 61-80), male sex, preshared care glaucoma scheme stages: Amber 2 (A2), Amber 3 (A3), Green 1 (G1), Green 2 (G2), Green 3 (G3), Red 1 (R1) and Red 2 (R2), and presence of interventions preshared care glaucoma scheme: peripheral iridotomy, selective laser trabeculoplasty, and any surgical intervention. A p value of <0.05 is generally considered statistically significant

Predictor variables	Coefficient	Standard error	95% confidence interval		z value	p value
			Lower limit	Upper limit		
Constant	0.09418	0.42250	-0.732	0.920	0.223	0.8236
Age group						
40-60 years	-1.07188	0.79563	-2.637	0.493	-1.347	0.1779
61-80 years	0.41614	0.33969	-0.249	1.081	1.225	0.2205
Male sex	-0.61956	0.33325	-1.272	0.033	-1.859	0.0630
A2	1.64187	1.14791	-0.625	3.908	1.430	0.1526
A3	-0.80200	1.23027	-3.225	1.621	-0.652	0.5145
G1	-0.88537	0.94610	-2.753	0.982	-0.936	0.3494
G2	-0.60891	0.54320	-1.681	0.463	-1.121	0.2623
G3	0.19024	0.48205	-0.755	1.135	0.395	0.6931
R1	0.33264	0.46343	-0.578	1.243	0.718	0.4729
R2	1.94265	1.05715	-0.122	4.008	1.838	0.0661
Peripheral iridotomy	-1.01565	0.74743	-2.479	0.447	-1.359	0.1742
Selective laser trabeculoplasty	0.02778	0.89881	-1.733	1.788	0.031	0.9753
Any surgical intervention	-0.63341	0.55651	-1.723	0.456	-1.138	0.2551

Summary of the key findings

Overall, the analysis found no significant deterioration in vision for all patients. No predictors were found to be significantly associated with deterioration in visual fields.

## Discussion

Shared care glaucoma scheme

During COVID-19, many countries faced challenges in glaucoma follow-up care, which led to worsening outcomes for this patient group. The challenges were mainly due to environmental factors such as transportation, hospital repurposing, risk of COVID-19 spread, and medication adherence [1-5]. Inevitably, this led to delays in glaucoma monitoring and care at HES [12]. Studies published in the United Kingdom and India found that due to the disruption in continuous glaucoma care, patients had a significant visual field deterioration, IOP elevation, and increased cup-to-disc ratios, leading to patients’ movement into the highest strata on the Glauc-Strat-Fast tool [12,13]. In response to the reduced appointment availability in HES, parts of the United Kingdom, including Lothian, had implemented the SCGS, which incorporates a local collaboration between NESGAT-qualified community optometrists and HES in managing glaucoma patients [14]. The main purpose of the scheme was to ensure glaucoma patients received appropriate continuity of care despite reduced HES appointments.

Assessing the SCGS in Lothian

This retrospective study of six years had similar proportions of male and female sex, comprising 85 men (49.4%) and 87 women (50.6%). Classification of the patients into their respective glaucoma stages based on the Glauc-Strat-Fast tool revealed stage R1, the most common stage in this study, followed by stages A1 and G3 [15]. In contrast to other studies investigating the effect of delayed HES appointments in this patient group, our study found that there was a minimal increase in IOP measurements in the post-SCGS phase compared to the pre-SCGS phase. Of the 16 patients (9.30%) who have been referred to the HES, no interventions were needed for 12 (6.98%) of them. The remaining four had interventions such as bilateral selective laser trabeculoplasty, bilateral peripheral iridotomies, unilateral trabeculectomy, and the addition of an IOP-reducing agent. Patients who were not referred did not experience a significant decline in MDVF or an increase in IOP, nor experienced significant ophthalmologic sequelae. A mild deterioration in MVDF was noted in the SCGS phase compared to the pre-SCGS phases (please quantify this if possible). Multiple regression analysis found no statistically significant association between the multitude of predictors and visual field deterioration.

Benefits of the SCGS

Like the SCGS, other countries have implemented alternative measures, such as telemetry and remote monitoring, to ensure the continuity of glaucoma follow-up during the pandemic. At-home tonometer devices and virtual reality perimetry were incorporated to replace the reduced in-person clinic spaces and travel restrictions [16-18]. Studies investigating these measures found that they were highly effective in glaucoma management and timely escalation to HES in cases of severe deterioration. This points to the possibility that the uninterrupted glaucoma follow-up provided by the SCGS potentially prevented severe disease deterioration in this patient cohort by continuous assessment of the IOP and visual field and ensuring medication adherence [10,19,20].

Limitations

Our study’s retrospective design was a limitation, which hindered a comprehensive assessment of all relevant predictors. While one community optometry confirmed the use of Goldmann applanation tonometry and Humphrey visual field analyzer, the instruments used for measurements were not confirmed in other community optometrists. This introduces the possibility of variation in IOP and MDVF measurements across the six community optometrists. Medication adherence in this patient cohort was not formally assessed and documented. Thus, it is unsure how this predictor can potentially affect disease management with the scheme. Another significant limitation was the huge proportion of patients who did not attend their clinic appointments at the community optometrist and HES. The factors contributing to noncompliance with clinic appointments were not assessed and documented, thus creating a bias toward only analyzing disease progression in patients compliant with clinic appointments. Furthermore, our sample size may have been insufficient in determining the impact on each of the subgroups, as stated in the Glauc-Strat-Fast tool. Finally, the nature of the condition, which progresses slowly over time, poses a challenge in the short period of the study. Therefore, our period of analysis may not be representative of the actual impact of the scheme in monitoring and assessing disease progression.

Future directions and research

Role of Community Optometrists

Future studies should assess the perspective of NESGAT-qualified optometrists on the scheme and the impact of glaucoma follow-up at community optometrists on waiting times for patients with other conditions. Additionally, confidence and factors affecting the confidence level of community optometrists in managing glaucoma patients should be assessed to allow a pragmatic approach if the scheme were to be implemented in the future.

Patient Factors

If the scheme were to be implemented in the future, noncompliance with clinic attendance should be studied, and measures should be taken to alleviate the hindering factors. Studies should also be done to investigate patient's perspectives on the scheme and their confidence in being followed up by a community optometrist. Measures should be taken to educate patients on the scheme, which will allow for better levels of compliance.

Implementing the SCGS

The scheme should be investigated further for a longer duration if it were to be implemented while incorporating other relevant variables such as medication adherence and ethnicity. Despite the lack of published studies on the scheme, there is potential to implement the scheme gradually, where the scheme begins with glaucoma patients of the Green and Amber groups of the Glauc-Strat-Fast tool. Alternatively, the Red group of the Glauc-Strat-Fast tool can be incorporated into the scheme but with shorter intervals between HES clinics compared to the other groups.

## Conclusions

In conclusion, there has been no significant deterioration in visual field values with SCGS implementation in Lothian, and disease stability was maintained in all subgroups of glaucoma patients. This finding is most likely explained by the continuous monitoring nature of the scheme, aided by the community optometrist appointment. The scheme thus allows for the appropriate escalation of patients requiring urgent care and frees up clinic space at HES. The study suggests that the period between HES clinic appointments can be prolonged, and the patients can be safely followed up by NESGAT-qualified community optometrists between the HES clinic appointments. A gradual launch approach should be used to introduce this scheme in the United Kingdom to allow a more systematic and efficient approach to monitoring glaucoma patients, preventing delays for both new and follow-up patients.
